# Predictive value and correlation analysis of neutrophil-to-lymphocyte ratio for early prognosis in patients with intracerebral hemorrhage undergoing surgical treatment

**DOI:** 10.3389/fneur.2026.1826034

**Published:** 2026-07-08

**Authors:** Hanmo Bao, Hui Wei, Yong Wang, Yajie Zhang, Wen Cai, Yuxuan Wu

**Affiliations:** 1 Neurocritical Care Unit, The Affiliated Hospital of Xuzhou Medical University, Xuzhou, Jiangsu, China; 2 Department of Cardiology, The Affiliated Hospital of Xuzhou Medical University, Xuzhou, Jiangsu, China

**Keywords:** inflammation, intracerebral hemorrhage, neutrophil-to-lymphocyte ratio, prognosis, surgical treatment

## Abstract

**Background:**

Intracerebral hemorrhage (ICH) represents a fatal acute neurological condition. Neutrophil-to-lymphocyte ratio’s (NLR) early prognostic value in surgical ICH needs validation.

**Objective:**

This study explored and quantified the early postoperative predictive value of NLR in surgically treated ICH patients.

**Methods:**

Retrospective data of 74 surgically treated ICH patients at The Affiliated Hospital of Xuzhou Medical University from November 2023 to September 2024 were collected, with 70 enrolled after exclusion. The primary endpoint was 30-day postoperative poor functional outcome [modified Rankin Scale (mRS) 4–6], and postoperative day 3 NLR (NLR-D3) was defined as the core predictive biomarker; secondary outcome was in-hospital complication incidence. All patients were stratified by 30-day mRS into good prognosis (mRS 0–3, *n* = 36) and poor prognosis (mRS 4–6, *n* = 34) groups. Spearman correlation analysis assessed NLR-prognosis correlation, receiver operating characteristic (ROC) curve analysis evaluated NLR predictive efficacy, and multivariate Logistic regression identified independent poor prognosis risk factors.

**Results:**

The poor prognosis group had older age, lower admission Glasgow coma scale scores, larger hematoma volumes, and higher rates of intraventricular extension and midline shift (all *p* < 0.05), along with significantly higher NLR at all pre-operative and postoperative time points (all *p* < 0.001), the most prominent difference was in postoperative day 3 NLR (NLR-D3). This group also had a higher in-hospital complication incidence, particularly pulmonary infection (all *p* < 0.05). NLR-D3 showed the highest correlation with 30-day mRS (r = 0.847); its AUC for poor prognosis prediction was 0.802 (95%CI: 0.682, 0.922), with an optimal cut-off of 8.6 (85.3% sensitivity, 83.3% specificity). Patients with NLR-D3 > 8.6 had a much higher poor prognosis rate (82.9% vs. 14.3%), and high NLR-D3 was confirmed as an independent risk factor for poor postoperative prognosis by multivariate analysis (*p* < 0.001).

**Conclusion:**

For surgically treated ICH patients, early postoperative elevated NLR, especially on day 3, correlates closely with adverse outcomes, acting as an inflammatory indicator for short-term poor prognosis.

## 1 Introduction

Intracerebral hemorrhage (ICH) is an acute neurological emergency characterized by persistently high morbidity, disability, and mortality. Accounting for 10 to 15% of all stroke cases, it affects over 4 million new patients worldwide annually. Within 30 days of onset, the mortality rate reaches 30 to 50%, and approximately 70% of survivors suffer from varying degrees of neurological deficits—imposing a heavy burden on patients’ families and the global healthcare system (1, 2). The pathophysiological process of ICH is highly complex. Beyond the primary compressive injury caused by the hematoma itself, a cascade of secondary events—triggered by hematoma degradation products—plays a pivotal role in exacerbating neurological dysfunction and shaping patient outcomes. These events include secondary inflammatory responses, cerebral edema, and disruption of the blood–brain barrier (3). Clinically, surgical hematoma evacuation to relieve intracranial hypertension remains the mainstay of ICH treatment. Yet, treatment efficacy varies drastically among individuals, influenced by factors such as baseline patient conditions, bleeding characteristics, surgical timing, and postoperative complications (4). Thus, identifying simple, reliable, and dynamically monitorable early prognostic indicators is crucial for recognizing high-risk patients, optimizing perioperative management strategies, and improving clinical outcomes.

Inflammation stands at the core of ICH pathophysiology. Following ICH, hematoma components activate microglia and astrocytes, while recruiting peripheral inflammatory cells—including neutrophils and lymphocytes—to infiltrate brain tissue. This triggers the release of massive inflammatory factors and chemokines, sparking a local inflammatory storm that aggravates cerebral edema and neuronal apoptosis (5). As the primary effector cells of innate immunity, neutrophils accumulate and activate rapidly in the early inflammatory phase, inducing secondary brain injury via elastase and reactive oxygen species release. Lymphocytes, by contrast, regulate adaptive immunity; their numerical reduction or functional impairment disrupts immune homeostasis, further amplifying inflammatory responses and tissue damage (6). The neutrophil-to-lymphocyte ratio (NLR), a simple inflammatory marker integrating changes in neutrophils and lymphocytes, indirectly reflects the degree of systemic inflammation and immune dysregulation. Boasting advantages of convenient detection, low cost, and dynamic monitorability, NLR has been widely applied in prognostic assessment for various acute critical illnesses and neurological disorders (7).

In recent years, growing attention has been paid to NLR’s value in evaluating ICH outcomes. Existing studies suggest that elevated NLR at admission correlates with poor prognosis in ICH patients, serving as a potential indicator for short-term mortality and neurological recovery. However, most research focuses on non-surgically treated patients or only assesses preoperative NLR’s predictive efficacy. For ICH patients undergoing surgery, the dynamic changes of early postoperative NLR and its prognostic value still require further clarification (8). Surgery itself elicits systemic stress responses, activating inflammatory cells and triggering inflammatory factor release—factors that may alter NLR trends. Meanwhile, postoperative complications such as pulmonary infection and intracranial infection can exacerbate inflammation, interfering with the association between NLR and prognosis (9). Preoperative NLR, therefore, fails to fully reflect postoperative inflammatory status and disease progression. Dynamically monitoring early postoperative NLR, and identifying its correlation with surgical outcomes as well as the optimal predictive time point, is essential for improving the prognostic assessment system for surgically treated ICH patients.

Current clinical indicators for ICH prognosis—including Glasgow Coma Scale (GCS) scores, hematoma volume, midline shift, and intraventricular extension—are mostly evaluated preoperatively, making them unable to dynamically reflect postoperative disease changes and inflammatory severity (10). As a dynamically monitorable laboratory index, NLR can real-time mirror postoperative systemic inflammation and immune function, potentially compensating for the limitations of traditional indicators (11, 12). Studies on neurological disorders such as ischemic stroke and traumatic brain injury have confirmed that early postoperative NLR exhibits excellent prognostic efficacy, with NLR values at specific time points showing superior predictive performance (13, 14). A recent large retrospective cohort study focusing on traumatic brain injury verified that elevated peripheral NLR was a powerful independent prognostic biomarker for in-hospital mortality and long-term neurological dysfunction in TBI patients; this cohort study systematically clarified the predictive efficiency of peripheral NLR for adverse neurological outcomes after acute traumatic brain injury, and further perfected the underlying neuroinflammatory pathological mechanism linking peripheral inflammatory indexes and secondary neuronal injury after acute structural brain damage (15). Nevertheless, consensus remains lacking regarding the dynamic changes of NLR at different postoperative time points in surgically treated ICH patients, which time point yields the best short-term prognostic value, and whether NLR constitutes an independent risk factor for poor outcomes—gaps that urgently require verification through clinical research.

Based on the aforementioned research gaps, this retrospective study collects clinical data of surgically treated ICH patients to explore the association between early postoperative NLR and 30-day neurological outcomes, clarifying its value in predicting poor prognosis. The study aims to provide a simple, reliable biological indicator for early identification of prognostic risks in surgically treated ICH patients, offer theoretical basis for optimizing perioperative management strategies (e.g., anti-inflammatory therapy and complication prevention), and ultimately improve patient clinical outcomes.

## 2 Patients and methods

### 2.1 Study design

**Figure 1:**
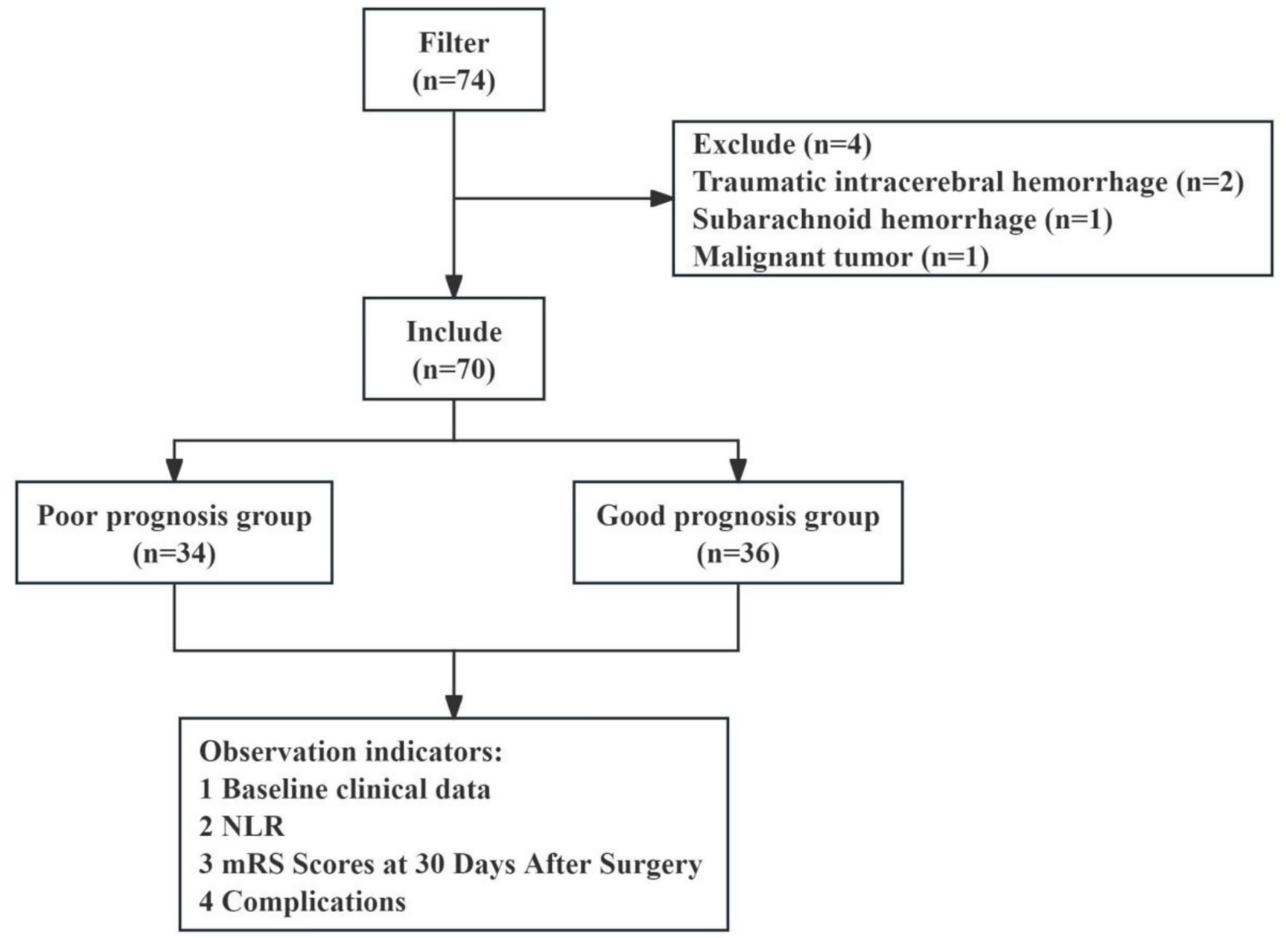
Research flowchart. Seventy-four patients were initially screened in this study, after exclusion, 70 cases were included, with 34 cases in poor prognosis group and 36 cases in good prognosis group. NLR, Neutrophil-to-Lymphocyte Ratio; mRS, modified Rankin Scale.

This retrospective study collected clinical data from 74 ICH patients who underwent surgical treatment at The Affiliated Hospital of Xuzhou Medical University between November 2023 and September 2024. After rigorous application of exclusion criteria, a total of 70 patients were finally enrolled in the study. All enrolled patients were stratified into two groups according to their modified Rankin Scale (mRS) scores at 30 days postoperatively: the favorable prognosis group (mRS 0–3, *n* = 36) and the poor prognosis group (mRS 4–6, *n* = 34) (Figure 1).

### 2.2 Inclusion criteria

(1) Diagnosed with spontaneous ICH, as confirmed by cranial CT or MRI. Imaging results clearly documented key information including bleeding site and hematoma volume; (2) Received surgical treatment in our hospital between November 2023 and September 2024. Surgical procedures covered craniotomy for hematoma evacuation, neuroendoscopic hematoma removal, and stereotactic hematoma puncture and drainage. All operations were performed in accordance with the surgical indications specified in the Chinese Guidelines for the Diagnosis and Treatment of Intracerebral Hemorrhage (2019 Edition) (16), and detailed surgical records (operation duration, surgical approach and intraoperative conditions) were fully preserved; (3) Hospital admission occurred within 72 h after symptom onset, with a clearly recorded time of onset, to ensure enrollment of patients with acute-phase ICH; (4) Complete preoperative and postoperative routine blood test results were available, including tests conducted on postoperative day 1, 3, and 5. These data allowed us to collect neutrophil and lymphocyte counts and calculate the NLR; (5) The 30-day postoperative modified Rankin Scale (mRS) score was recorded. Patients were divided into the favorable prognosis group (mRS 0–3) and poor prognosis group (mRS 4–6) based on the score (17). Information on in-hospital complications such as pulmonary infection, rebleeding and intracranial infection was also fully documented; (6) Complete medical records could be retrieved for analysis; (7) The patient was at least 18 years of age.

### 2.3 Exclusion criteria

(1) Secondary hemorrhagic diseases, such as traumatic ICH, subarachnoid hemorrhage, simple intraventricular hemorrhage, other cerebrovascular malformations, or tumor rupture; (2) Patients with severe ongoing infectious diseases, autoimmune disorders, malignant neoplasms, chronic liver disease or chronic kidney disease were excluded. These conditions are known to alter peripheral neutrophil counts, lymphocyte counts and the NLR; (3) History of long-term glucocorticoid or immunosuppressant use before admission, or acute inflammatory response within the recent month; (4) Presence of mental disorders or cognitive impairment (18).

### 2.4 Data collection

Study data were independently extracted and entered by two uniformly trained physicians. Discrepancies were reviewed and resolved by a third senior physician, with a cross-verification consistency rate of ≥ 95%. Collected data included demographic information (gender, age), history of underlying diseases (hypertension, diabetes), preoperative clinical characteristics (GCS score, systolic blood pressure [SBP], time from onset to surgery, surgical approach), preoperative imaging indicators (bleeding location, hematoma volume, intraventricular extension, degree of midline shift), laboratory indicators (neutrophil count, lymphocyte count, and calculated NLR before surgery and on postoperative days 1, 3, and 5), surgical records, and incidence of in-hospital complications (pulmonary infection, rebleeding, intracranial infection). Outcome indicators were obtained through medical record review: the primary endpoint was 30-day postoperative poor functional outcome (mRS 4–6), postoperative day 3 NLR (NLR-D3) was defined as the core predictive biomarker of primary endpoint, and the secondary outcome was the incidence of in-hospital complications. The 30-day postoperative total mRS score was adopted for patient prognosis grouping. Data were confirmed to be accurate, complete, and consistent before being used for subsequent statistical analysis.

### 2.5 Observation indicators

1. SBP: Measured on the patient’s right upper arm using an electronic sphygmomanometer (HEM-7200, Omron Healthcare Co., Ltd., Japan);
2. Preoperative imaging indicators: Scanned using a magnetic resonance imager (Ingenia 3.0 T, Philips Healthcare Systems, the Netherlands) and independently interpreted by two radiologists. Bleeding location was recorded by anatomical region; hematoma volume was calculated using the Tada formula (Volume = *π*/6 × length × width × height) (19, 20). Intraventricular extension and degree of midline shift (maximum displacement distance of midline structures) were determined based on scan images;
3. GCS score: Manually scored based on eye-opening, verbal, and motor responses, with a total score ranging from 3 to 15 — lower scores indicate more severe coma (21);
4. Neutrophil count, lymphocyte count, and NLR: Peripheral blood samples were collected before surgery and on postoperative days 1, 3, and 5. Neutrophil and lymphocyte counts were detected using an automatic hematology analyzer (DxFLEX, Beckman Coulter, Inc., USA), and NLR was calculated as (neutrophil count / lymphocyte count) (22);
5. mRS score: Focused on evaluating the patient’s overall functional status and independence in daily life. The mRS score ranges from 0 to 6 (7 grades): 0 = no symptoms; 1 = symptoms present but no significant disability; 2 = mild disability; 3 = moderate disability; 4 = moderately severe disability; 5 = severe disability; 6 = death (23).
6. Postoperative complications: Pulmonary complications: hospital-acquired pneumonia, pleural effusion, atelectasis. Intracranial complications: postoperative rebleeding, intracranial infection (meningitis/ventriculitis), hydrocephalus, cerebral vasospasm. Systemic complications: urinary tract infection, gastrointestinal bleeding, liver and renal function impairment, electrolyte disturbance. Other adverse events: deep vein thrombosis, pressure sore.

### 2.6 Ethical statement

This study has been approved by the Medical Ethics Committee of the Affiliated Hospital of Xuzhou Medical University, and informed consent was obtained from all participants (Approval No.: XYFY2024-KL219-01). The study was conducted in strict compliance with the Declaration of Helsinki.

### 2.7 Sample size calculation

No prospective sample size calculation was performed in this study. The final sample size of 70 cases was determined based on the number of patients who met the inclusion criteria and had complete clinical data. A previous single-center study (24), reported significant changes in the neutrophil-to-lymphocyte ratio (NLR) in patients with intracerebral hemorrhage (ICH) before surgery and 3 days after surgery (3.7 [2.4–6] vs. 4.6 [3.3–6.2], *p* < 0.001). The calculated Cohen’s D was 0.62. With a two-sided test set at *α* = 0.05 and *β* = 0.20, the required sample size for each group was estimated to be 33 patients. The actual sample size in our study (36 patients in the favorable prognosis group and 34 in the poor prognosis group) exceeded the estimated value, indicating an adequate statistical power (>80%). This suggests that the present study has sufficient sensitivity to detect the target effect.

### 2.8 Statistical analysis

Logistic regression was used to control for confounding biases in baseline data. Quantitative data were first subjected to normality test (Shapiro–Wilk test) and homogeneity of variance test (Levene test). Data conforming to normal distribution and homogeneous variance were expressed as mean ± standard deviation (mean ± SD), with independent samples *t*-test for intergroup comparison and paired *t*-test for intragroup comparison. Non-normally distributed quantitative data were expressed as median (interquartile range) [M (Q1, Q3)], with Mann–Whitney *U*-test for intergroup comparison and Wilcoxon rank-sum test for intragroup comparison. Categorical clinical indicators were summarized as case count plus constituent percentage [*n*(%)], with chi-square test applied for between-group comparison; whenever expected cell frequencies fell below five, Fisher’s exact test replaced conventional chi-square algorithms instead. Spearman rank correlation analysis was used to explore the correlation between NLR and ICH prognosis. Indicators with *p* < 0.05 in univariate analysis were included in the Logistic regression model to screen for independent risk factors for poor ICH prognosis. Receiver operating characteristic (ROC) curves were plotted to evaluate the predictive efficacy of the NLR model, with calculation of the area under the curve (AUC), sensitivity, and specificity. All statistical tests were two-tailed, and a *p*-value <0.05 was considered statistically significant.

## 3 Results

### 3.1 Baseline data

A comparison of baseline clinical data between the poor prognosis group and favorable prognosis group among surgically treated ICH patients revealed significant statistical differences in age, admission GCS score, hematoma volume, incidence of intraventricular extension (58.8% vs. 25.0%), and degree of midline shift (all *p* < 0.05). No statistically significant differences were observed between the two groups in terms of gender, body mass index, prevalence of hypertension and diabetes, admission SBP, time from onset to surgery, or distribution of surgical approaches (all *p* > 0.05) (Table 1). These findings provide a baseline basis for further screening of independent risk factors for poor prognosis.

**Table 1:** Baseline clinical data.

Indicators	Poor prognosis group (*n* = 34)	Good prognosis group (*n* = 36)	*p*	OR	95%CI for OR
Age (years, mean ± SD)	70.15 ± 7.26	62.92 ± 5.64	<0.001	0.843	0.770,0.924
Gender (*n* %)					
Male	21 (61.8)	20 (55.6)	0.598	1.292	0.498,3.356
Female	13 (38.2)	16 (44.4)			
BMI (kg/m2, mean ± SD)	24.54 ± 2.17	24.65 ± 1.54	0.795	1.034	0.802,1.334
Hypertension (*n* %)	27 (79.4)	22 (61.1)	0.099	2.455	0.844,7.140
Diabetes (*n* %)	14 (41.2)	10 (27.8)	0.240	1.820	0.670,4.943
GCS (scores, mean ± SD)	7.64 ± 2.37	12.50 ± 2.17	<0.001	2.568	1.662,3.968
SBP (mmHg, mean ± SD)	158.71 ± 18.05	155.83 ± 11.59	0.423	0.987	0.956,1.019
TOS (h, mean ± SD)	9.24 ± 2.67	8.67 ± 2.21	0.326	0.906	0.745,1.103
Surgical procedure (*n* %)					
Craniotomy for hematoma evacuation	18 (52.9)	16 (44.4)	0.478	1.406	0.549, 3.604
Endoscopic hematoma evacuation	9 (26.5)	12 (33.3)	0.532	1.389	0.496, 3.890
Stereotactic puncture and drainage	7 (20.6)	8 (22.2)	0.868	1.102	0.351, 3.459
Hematoma volume (mL, mean ± SD)	42.12 ± 10.15	25.07 ± 7.21	<0.001	0.791	0.708,0.885
Intraventricular rupture (*n* %)	20 (58.8)	9 (25.0)	0.005	4.286	1.549,11.857
Degree of midline shift (mm, mean ± SD)	4.97 ± 1.49	1.93 ± 0.97	<0.001	0.096	0.028,0.324

OR, Odds Ratio; 95%CI, 95% Confidence Interval; BMI, Body Mass Index; SBP, Systolic Blood Pressure; GCS, Glasgow Coma Scale; TOS, Time Onset to Surgery.

### 3.2 NLR

**Figure 2:**
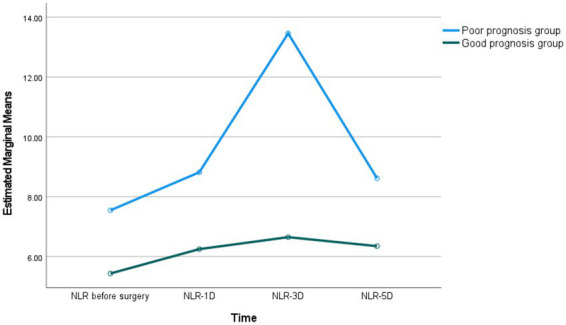
Trends in NLR.

Figure 2 illustrates the dynamic changes in NLR among surgically treated ICH patients in both groups before surgery and on postoperative days 1, 3, and 5. Results showed that NLR levels in the poor prognosis group were significantly higher than those in the favorable prognosis group at all time points (all *p* < 0.05). Meanwhile, both groups exhibited a consistent NLR variation trend: increasing from preoperatively to postoperative day 3, then declining from postoperative day 3 to day 5. Notably, the 3rd postoperative day showed the maximum intergroup difference in NLR levels, suggesting that NLR on postoperative day 3 (NLR-D3) could serve as a key time point for distinguishing patient prognosis. The interaction of group and time was statistically significant (*F* = 67.370, *p* < 0.001), with a partial eta squared of 0.498 (Table 2). The poor prognosis group exhibited significantly higher NLR values than the good prognosis group at baseline, postoperative day 1, day 3 and day 5 (all *p* < 0.001) (Table 3).

**Table 2:** Interaction between group and time.

Indicators	*F*	*p*	Partial eta sauared
Group*Time	67.370	<0.001	0.498

**Table 3:** Between-group comparison of NLR.

Indicators	Poor prognosis group (*n* = 34)	Good prognosis group (*n* = 36)	Z	*p*
NLR before surgery	7.65 (6.78, 8.83)	5.45 (4.53, 6.38)	−5.289	<0.001
NLR-1D	9.05 (8.25, 9.93)	6.25 (5.33, 7.18)	−5.053	<0.001
NLR-3D	13.40 (11.75, 15.25)	6.65 (5.73, 7.58)	−7.192	<0.001
NLR-5D	8.85 (7.98, 9.73)	6.35 (5.43, 7.28)	−5.018	<0.001

NLR, Neutrophil-to-Lymphocyte Ratio.

### 3.3 Incidence of complications

In the poor prognosis group, the incidences of pulmonary infection, intracranial infection, and rebleeding were 41.2, 11.8, and 14.7% respectively, with the total complication rate reaching 67.6%. For the favorable prognosis group, the corresponding rates were 16.7, 2.8, and 2.8%, and the total complication rate was only 22.2% (Table 4). These results indicate significant statistical differences between the two groups in the incidence of pulmonary infection (*p* = 0.023) and total complications (*p* < 0.001), suggesting that patients in the poor prognosis group had a substantially higher risk of developing complications.

**Table 4:** Comparison of complications.

Indicators	Pulmonary infection (*n* %)	Intracranial infection (*n* %)	Rebleeding (*n* %)	Total (*n* %)
Poor prognosis group (*n* = 34)	14 (41.2)	4 (11.8)	5 (14.7)	23 (67.6)
Good prognosis group (*n* = 36)	6 (16.7)	1 (2.8)	1 (2.8)	8 (22.2)
Phi	0.271	<0.001	0.213	0.457
*P*	0.023	1.000	0.075	<0.001

### 3.4 Correlation between NLR and 30-day postoperative mRS score

Table 5 analyzes the correlation between NLR (measured preoperatively and at different postoperative time points) and 30-day postoperative mRS score in surgically treated ICH patients. Results demonstrated that NLR values at all time points—preoperatively, and on postoperative days 1, 3, and 5—were significantly positively correlated with the 30-day postoperative mRS score (all *p* < 0.001). The Spearman correlation coefficients (r) were 0.664, 0.650, 0.847, and 0.682 in sequence. Notably, NLR-3D yielded the highest correlation coefficient with the 30-day postoperative mRS score, indicating the strongest association between NLR at this time point and the degree of neurological deficit in patients 30 days after surgery.

**Table 5:** Correlation between NLR and 30-Day postoperative mRS scores.

Indicators		Spearman correlation (*r*)	*p*
mRS scores at 30 days after surgery	NLR before surgery	0.664	<0.001
	NLR-1D	0.650	<0.001
	NLR-3D	0.847	<0.001
	NLR-5D	0.682	<0.001

NLR, Neutrophil-to-Lymphocyte Ratio.

### 3.5 Predictive efficacy of NLR-D3

**Figure 3:**
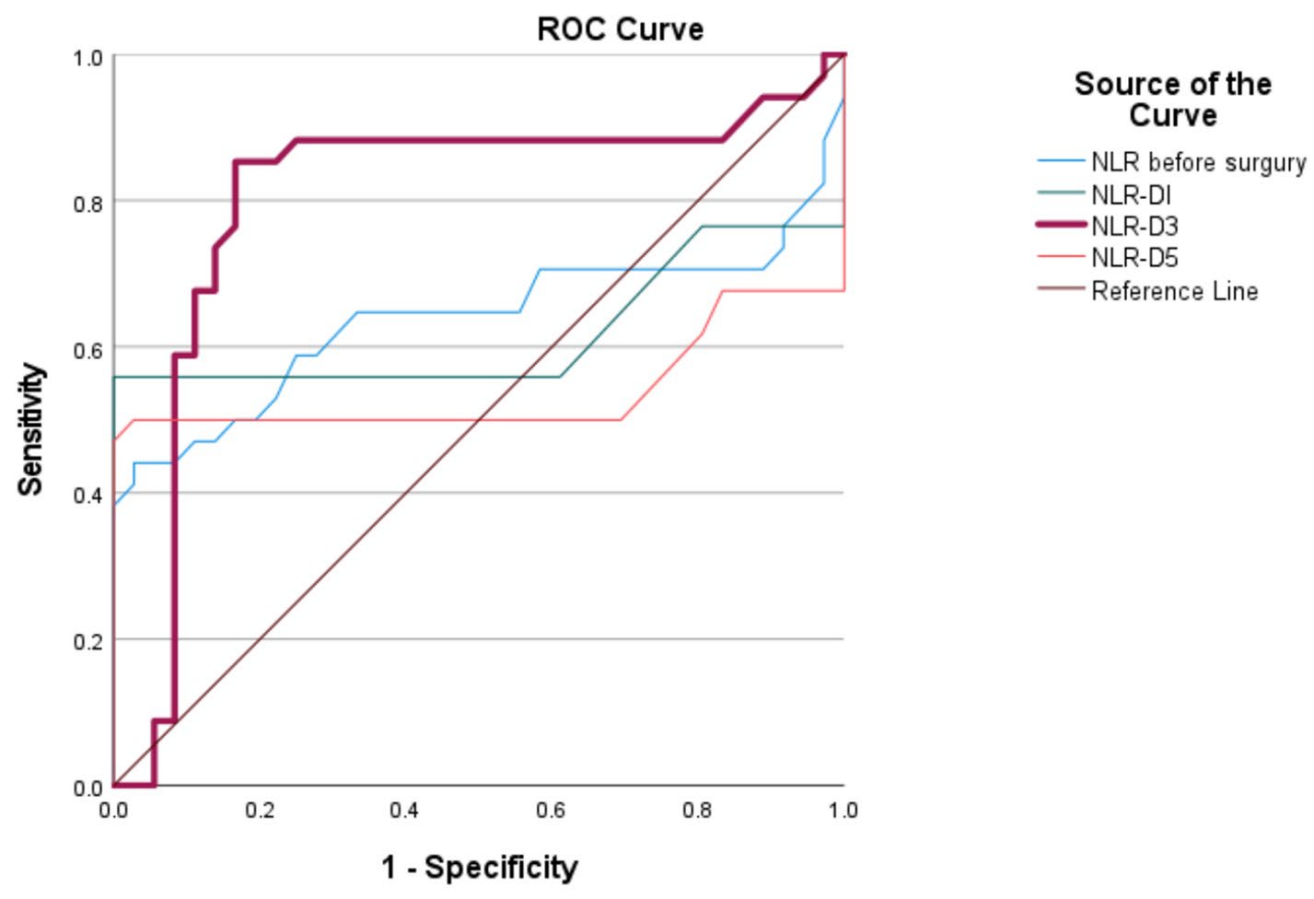
Predictive efficacy of NLR-D3. ROC, Receiver Operating Characteristic.

Figure 3 presents the ROC curves of NLR measured at different time points for predicting poor 30-day postoperative prognosis in surgically treated ICH patients. Table 6 summarizes the corresponding diagnostic performance indicators. Among all time points, NLR-D3 yielded the highest AUC of 0.802 (95% CI: 0.682, 0.922). With the optimal cut-off value of 8.6, the sensitivity and specificity were 85.3 and 83.3%, respectively. The Hosmer-Lemeshow test returned a *p* value of 0.811, indicating good model fit. DeLong test was adopted to compare AUCs across time points (using NLR-D3 as the reference). The AUC of preoperative NLR (AUC = 0.637, *p* = 0.034), NLR-D1 (AUC = 0.619, *p* = 0.047) and NLR-D5 (AUC = 0.540, *p* = 0.009) were all significantly lower than that of NLR-D3. These results demonstrate that NLR detected on postoperative day 3 has the best predictive ability for poor 30-day prognosis. The cut-off value of 8.6 for NLR-D3 was calculated and validated within the same small study cohort. Since neither internal nor external validation was performed, this result is presented solely as an exploratory analysis.

**Table 6:** Diagnostic efficacy indicators of ROC curve.

Indicators	Cut-off	Sensitivity (%)	Specificity (%)	AUC	95%CI for AUC	Hosmer-Lemeshow (*P*)	Delong (*p*)
NLR before surgery	6.35	58.8	75.0	0.637	0.493, 0.781	0.153	0.034
NLR-D1	7.25	55.9	77.8	0.619	0.468, 0.770	0.256	0.047
NLR-D3	8.6	85.3	83.3	0.802	0.682,0.922	0.811	-
NLR-D5	8.4	50.0	72.0	0.540	0.381, 0.698	0.333	0.009

ROC, Receiver Operating Characteristic; AUC, Area Under the Curve. Delong (P), vs NLR-D3.

### 3.6 Comparison of poor prognosis incidence between patients with different NLR-D3 levels

The cut-off value of 8.6 for NLR-D3 was calculated and validated within the same small study cohort. Since neither internal nor external validation was performed, this result is presented solely as an exploratory analysis. Based on the optimal cut-off value of 8.6 for NLR-D3, 70 surgically treated ICH patients were divided into the high NLR group (>8.6, *n* = 35) and the low NLR group (≤8.6, *n* = 35). Results in Table 7 showed that the incidence of poor 30-day postoperative prognosis in the high NLR group was 82.9%, which was significantly higher than the 14.3% in the low NLR group (*p* < 0.001). This indicates that patients with NLR-D3 levels exceeding 8.6 have a markedly increased risk of poor 30-day postoperative prognosis.

**Table 7:** Incidence of poor prognosis in patients with different NLR-D3 levels.

Indicators	Poor prognosis
High NLR group (>8.6) (*n* = 35)	29 (82.9%)
Low NLR group (≤8.6) (*n* = 35)	5 (14.3%)
*p*	<0.001
Phi	0.686

### 3.7 Independent risk factors for poor prognosis

To identify the independent risk factors for poor 30-day postoperative prognosis in surgically treated ICH patients, indicators with statistical significance in univariate analysis were included in the multivariate Logistic regression analysis. Results in Table 8 demonstrated that after adjusting for confounding factors such as age, admission GCS score, hematoma volume, intraventricular extension, and degree of midline shift, high NLR-D3 was an independent risk factor for poor 30-day postoperative prognosis (B = 2.176, *p* < 0.001, OR = 8.824, 95% CI: 3.105, 25.069). Other indicators, including age, admission GCS score, and hematoma volume, showed no statistical significance after adjustment (all *p* > 0.05). These findings suggest that high NLR-D3 possesses independent predictive value for poor postoperative prognosis in such patients. Given the limited sample size and exploratory nature of this study, we did not further construct a baseline clinical model to assess the incremental predictive value of NLR-D3. Such model comparison requires a larger number of outcome events to avoid overfitting.

**Table 8:** Analysis of independent risk factors.

Indicators	*B*	*p*	OR	95% CI for OR	Bootstrap-corrected OR	Bootstrap 95% CI for OR
Age (year)	0.021	0.256	1.021	0.985,1.059	1.019	0.982, 1.061
GCS (score)	−0.089	0.183	0.915	0.812,1.031	0.918	0.809, 1.035
Hematoma volume (mL)	0.015	0.167	1.015	0.992,1.039	1.014	0.990, 1.041
Intraventricular rupture	0.528	0.235	1.695	0.687,4.182	1.682	0.671, 4.215
Degree of midline shift (mm)	0.063	0.312	1.065	0.958,1.184	1.062	0.954, 1.189
NLR-D3	2.176	<0.001	8.824	3.105,25.069	8.675	2.983, 25.426

## 4 Discussion

This retrospective study analyzed clinical data from 70 surgically treated ICH patients, aiming to explore the predictive value of early postoperative NLR for short-term patient prognosis. Baseline data comparison revealed that the poor prognosis group had older age, lower GCS scores, larger hematoma volumes, higher incidences of intraventricular extension, and more severe midline shift. Dynamic NLR monitoring showed that NLR levels in the poor prognosis group were significantly higher than those in the favorable prognosis group at all time points—preoperatively, and on postoperative days 1, 3, and 5. Among these, NLR-D3 exhibited the most prominent intergroup difference and the highest correlation coefficient with the 30-day postoperative mRS score. ROC curve analysis confirmed favorable efficacy of NLR-D3 in predicting poor prognosis, with patients having high NLR-D3 levels showing a higher incidence of poor outcomes than those with low levels. Multivariate Logistic regression analysis further identified high NLR-D3 as an independent risk factor for poor 30-day postoperative prognosis. Additionally, the poor prognosis group had a higher total in-hospital complication rate and pulmonary infection rate, further supporting the association between elevated NLR and disease severity as well as adverse outcomes.

This study confirmed a close correlation between NLR-D3 and short-term postoperative prognosis of ICH. As an acute critical illness of the central nervous system, ICH pathogenesis involves not only primary compressive injury caused by the hematoma itself but also cascade inflammatory responses triggered by hematoma degradation products. As a simple indicator reflecting systemic inflammatory response and immune function, NLR can effectively integrate dynamic changes in neutrophils and lymphocytes, indirectly reflecting the degree of systemic inflammatory imbalance (25, 26). Neutrophils, as core effector cells of inflammation, may be activated by hematoma components and oxidative stress products after ICH, extensively infiltrating brain tissue and releasing proteases, reactive oxygen species, and pro-inflammatory cytokines. This exacerbates blood–brain barrier disruption, cerebral edema, and neuronal apoptosis; meanwhile, neutrophil activation may induce systemic inflammatory response syndrome, further worsening the condition (27). Lymphocytes, by contrast, act as immune regulatory cells—under inflammatory stress, they are prone to increased apoptosis and functional inhibition, leading to immune dysfunction that fails to effectively regulate inflammatory responses. This forms a vicious cycle of excessive inflammatory activation and immune suppression, a process that presumably peaks on postoperative day 3 (24), consistent with the most significant intergroup difference in NLR-D3 observed in this study. The higher proportion of elderly patients in the poor prognosis group may amplify NLR’s prognostic value by influencing inflammatory responses. Physiological age-related decline in immune function leads to reduced lymphocyte proliferation and differentiation capacity, while neutrophils maintain relatively enhanced reactivity to inflammatory stimuli—rendering inflammatory imbalance more pronounced and NLR elevation more significant after ICH (28, 29). Additionally, elderly patients often have comorbid underlying diseases, increasing the risk of postoperative complications, which in turn exacerbate inflammation and disease progression, forming a vicious cycle. Furthermore, the poor prognosis group’s lower admission GCS score, larger hematoma volume, and higher rates of intraventricular extension and midline shift all indicate more severe initial conditions. Such patients tend to experience more intense postoperative inflammatory responses, resulting in higher NLR levels. As an indicator of the inflammatory peak, NLR-D3 can more accurately reflect disease severity and systemic compensatory capacity (30), hence its superior predictive efficacy compared to NLR values preoperatively and on postoperative days 1 and 5. Intergroup differences in complication rates may also be closely linked to NLR levels, particularly for pulmonary infection. Postoperative ICH patients are at high risk of pulmonary infection due to impaired consciousness, weakened cough reflex, and bed rest. In a high NLR state, excessive neutrophil activation may increase airway mucus secretion and induce immune dysfunction, further reducing pulmonary anti-infective capacity. Simultaneously, systemic inflammatory responses may aggravate lung tissue damage, promoting pulmonary infection (31, 32). Conversely, secondary inflammatory responses triggered by pulmonary infection can further elevate NLR, forming a bidirectional vicious cycle—this may explain why the poor prognosis group had significantly higher incidences of pulmonary infection and total complications than the favorable prognosis group.

A representative large retrospective ICH cohort study (33) demonstrated that elevated peripheral NLR was an independent adverse prognostic biomarker for spontaneous intracerebral hemorrhage, and baseline NLR exhibited stable predictive performance for 30-day and 90-day poor functional outcomes in ICH patients across the entire large cohort, which provided high-level cohort evidence for the clinical application of NLR in ICH prognostic evaluation. A multicenter study (34) showed that NLR has the best predictive value for stroke-associated pneumonia in ICH patients (AUC: 0.748, 95%CI: 0.695–0.801), which is consistent with the findings of this study. A meta-analysis (35) confirmed that high NLR is significantly associated with adverse outcomes in ICH patients (*p* < 0.001). The CLEAR III analysis (36) also reported a correlation between NLR-D3 and 30-day postoperative infections in ICH patients, aligning with our results. A retrospective study (37) found that NLR at 48 h postoperatively predicted 30-day ICH prognosis with an AUC of 0.785 and an optimal cut-off value of 7.9—slightly lower than NLR-D3 and its cut-off value of 8.6 in this study—and identified 48-h postoperative NLR as an independent risk factor. This discrepancy may stem from differences in baseline characteristics of study populations: the mean age of patients in that study was 65.3 years, lower than the 70.15 years in the poor prognosis group of this study, with variations in comorbidity rates. Inflammatory response patterns in elderly patients may differ from those in young and middle-aged patients, leading to differences in NLR peak timing and cut-off values.

The innovations of this study lie in focusing on dynamic monitoring of early postoperative NLR and confirming NLR-D3 as the optimal time point for predicting short-term prognosis in surgically treated ICH patients. Most existing studies only compare NLR at a single preoperative and postoperative time point, whereas this study systematically monitored NLR changes preoperatively and on postoperative days 1, 3, and 5. Correlation analysis confirmed the strongest association between NLR-D3 and 30-day postoperative mRS score, and ROC curve analysis verified its superior predictive efficacy—providing a basis for clinical selection of accurate monitoring time points. Additionally, this study included baseline risk factors such as age, admission GCS score, hematoma volume, intraventricular extension, and midline shift in multivariate analysis. After adjustment, high NLR-D3 remained the only independent risk factor, excluding interference from baseline disease severity on prognostic prediction and confirming the independent clinical value of NLR-D3 as an inflammatory indicator, rather than merely a concomitant marker of disease severity.

## 5 Study limitations and future research directions

This study has certain limitations that need to be addressed in subsequent research. First, as a single-center retrospective analysis, it has a small sample size (*n* = 70) and a narrow inclusion time window (November 2023 to September 2024), which may introduce selection bias. The poor prognosis group is concentrated in the elderly age range of 55–84 years, lacking sufficient data on young and middle-aged patients, which limits the external validity of the results. Future studies should conduct multi-center, large-sample prospective cohort studies, including surgically treated ICH patients of different age groups and bleeding locations, to further verify the predictive efficacy of NLR-D3 and the stability of its cut-off value. Second, this study only monitored NLR as a single inflammatory indicator, without simultaneous detection of other inflammatory markers such as C-reactive protein and procalcitonin. Thus, the combined predictive value of NLR with other indicators cannot be clarified. Future research may explore multi-indicator combined prediction models to further improve the accuracy of prognostic prediction. Third, this study did not analyze the impact of different therapeutic interventions on NLR and the effect of prognosis improvement. Prospective interventional studies are needed in the future to explore the influence of individualized treatment regimens targeting NLR on the prognosis of surgically treated ICH patients. Fourth, this study focused on short-term prognosis at 30 days postoperatively, with no long-term follow-up of patients. Consequently, the predictive value of NLR-D3 for long-term neurological function recovery remains unclear. Subsequent studies should extend the follow-up period to improve the analysis of the association between NLR and long-term prognosis. Fifth, this study did not consider the interference of preoperative immune function status and concurrent medication history on NLR—factors that may affect NLR levels and inflammatory response patterns. Future research should strictly control such confounding factors to further optimize study design. Sixth, no external data validation was performed in this study. Multi-center, large-sample prospective studies are still required to further verify its value in clinical promotion and application. Finally, due to the small sample size and insufficient adverse outcome events, we failed to evaluate the incremental predictive value of NLR-D3 by comparing the baseline clinical model and the combined model. This is a retrospective exploratory study, and no *a priori* power analysis was performed. Large-sample multicenter studies are needed in the future to verify the incremental predictive effect of NLR-D3 on the basis of conventional clinical predictors. The presence of complications such as infection and rebleeding may interfere with the predictive value of NLR. We will include these confounding factors for analysis in future studies.

## 6 Conclusion

As a simple and repeatable inflammatory indicator, NLR can be used for early postoperative prognostic assessment in surgically treated ICH patients. Dynamic monitoring of NLR, especially NLR-D3, helps clinicians identify high-risk patients early and adjust perioperative management strategies in a timely manner, thereby improving patient clinical outcomes. Future multi-center prospective studies are needed for further verification to promote the widespread application of NLR in the clinical management of ICH.

## Data Availability

The original contributions presented in the study are included in the article/supplementary material, further inquiries can be directed to the corresponding author.
